# College and Hospital Notes

**Published:** 1904-06

**Authors:** 


### COLLEGE AND HOSPITAL NOTES.

HOSPITAL APPOINTMENTS.—The following-named graduates in medicine of the University of Buffalo, class of 1904, have received appointments as internes:

*Buffalo General Hospital.*

Frederick S. Brickell,
Melvil S. Coxe,
Claude S. Johnson,
L. Kauffman,
Edward C. Koenig,
Harry R. Lohnes,
Victor M. Rice,
Floyd Richardson,
Robert F. Sheehan, Jr.,
Herbert N. Squier.

*Sisters of Charity Hospital.*

Edward J. Durney,
Thomas F. Foley,
William S. Lynch.

*Erie County Hospital.*

Parker G. Borden,
John F. Fairbairn,
Chauncey W. Grove,
John G. Morris,

*Erie County Hospital—Continued.*

Douglass H. Smith,
John L. Van DeMark,
Glenn L. Whiting.

*Emergency Hospital.*

John H. Burke,
John C. Plain,
Raymond A. Turnbull.

*German Hospital.*

Robert C. Mehnert,
Samuel A. Moore,
Herman W. Schlappi.

*Deaconess's Hospital.*

Leonard Reu,
Julius Richter,
Charles W. Selover.

*Rochester City Hospital.*

Halley W. Hammond.

The Buffalo General Hospital has issued its forty-fifth annual report which covers the year 1903, and which is by far the most complete it has ever sent out. The president of the board of trustees, Mr. Stephen M. Clement, makes a very clear and interesting statement of the affairs of the hospital, which betrays the business man and the financier. He speaks of the debt which has been cleared off during the year, mention of which has been made heretofore in the Journal. The hospital, however, is still without proper endowment, about $200,000 additional being necessary.

The first training school for nurses west of New York City was founded in this hospital in 1877, since which time 271 nurses have been graduated. A new nurses' home is building which will accommodate 64 inmates, thus adding a much needed improvement to this service. During 1903, 2,635 patients were treated at an expense of $1.47 a day for each individual. The report is handsomely printed and well illustrated.

